# Genetically instrumented circulating metabolites and hepatobiliary cancer risk: A multi-tiered Mendelian randomization and functional interrogation

**DOI:** 10.3389/fonc.2025.1680865

**Published:** 2025-10-27

**Authors:** Lin Tuo, Li Ting Yan, Ying Liu, Shu Qiang Wang, Xing Xiang Yang, Xiang An

**Affiliations:** ^1^ Department of Infectious Disease, Sichuan Provincial People’s Hospital, University of Electronic Science and Technology of China, Chengdu, China; ^2^ Department of Hepatobiliary and Pancreatic Surgery, Sichuan Provincial People’s Hospital, University of Electronic Science and Technology of China, Chengdu, China

**Keywords:** circulating metabolites, multivariable mendelian randomization analysis, hepatobiliary malignancies, 4-hydroxyhippurate, 3-hydroxyisobutyrate

## Abstract

**Background:**

Hepatobiliary malignancies—including hepatocellular carcinoma and cholangiocarcinoma—are major causes of cancer-related mortality worldwide, yet their regulatory pathways remain incompletely defined.

**Methods:**

We employed a two-sample Mendelian randomization (MR) approach to systematically investigate causal relationships between 1,400 serum metabolites and hepatobiliary cancer risk. Through stringent quality control (all SNPs with F-statistics > 10) and sensitivity analyses (MR-Egger regression, weighted median method, and MR-PRESSO), we identified 10 candidate metabolites.

**Results:**

Meta-analysis confirmed three metabolites with robust associations: risk-increasing dimethylarginine (SDMA+ADMA) and 4-hydroxyhippurate, and protective 3-hydroxyisobutyrate. Multivariable MR validated the independent effects of 4-hydroxyhippurate and 3-hydroxyisobutyrate. In vitro functional experiments demonstrated that 4-hydroxyhippurate promoted, whereas 3-hydroxyisobutyrate inhibited, hepatocellular carcinoma cell proliferation.

**Conclusion:**

These findings advance understanding of metabolic dysregulation in hepatobiliary malignancies and nominate candidate diagnostic biomarkers and therapeutic targets, providing translationally relevant hypotheses for precision medicine.

## 1 Introduction

Hepatobiliary malignancies—including hepatocellular carcinoma (HCC) and cholangiocarcinoma (CCA)—account for a rising share of global cancer mortality, with marked geographic variation driven by viral hepatitis, metabolic dysfunction–associated steatotic liver disease, alcohol, aflatoxin exposure, and primary sclerosing cholangitis (1–3). Despite advances in surveillance and therapy, late-stage presentation and molecular heterogeneity continue to limit outcomes (4, 5).

Metabolic reprogramming is a hallmark of hepatobiliary tumorigenesis (6, 7). Perturbations have been reported across amino-acid metabolism (e.g., branched-chain and aromatic amino acids), one-carbon/arginine–NO pathways (including asymmetric and symmetric dimethylarginine), short-chain and hydroxy–carboxylic acids (e.g., 3-hydroxyisobutyrate from valine catabolism), bile acid and lipid remodeling, and host–microbiome co-metabolites such as hippurate derivatives (e.g., 4-hydroxyhippurate) (8–10). Several case–control and prospective metabolomics studies suggest associations of these metabolites with HCC/CCA risk or progression, yet effect directions and specificity vary across platforms, biospecimens, and populations (11–13).

Observational associations between circulating metabolites and cancer risk are prone to confounding (e.g., lifestyle, liver function, inflammation) and reverse causation due to subclinical disease (14). Mendelian randomization (MR) leverages germline variants as instruments to strengthen causal inference under three assumptions (relevance, independence, exclusion restriction) and has been increasingly applied to metabolic traits. Recent mGWAS provide strong instruments for hundreds of metabolites, enabling two-sample MR while minimizing sample overlap and enhancing generalizability (15, 16).

To address uncertainties from observational metabolomics, we aimed to systematically evaluate the potential causal effects of 1,400 circulating metabolites and ratios on the risk of hepatobiliary cancers using a two-sample MR framework. Specifically, we prespecified a discovery–replication design across independent outcome GWAS (FinnGen; UK Biobank via Neale lab/IEU), applied stringent instrument selection, harmonization, and robustness analyses (IVW, MR-Egger, weighted median/mode, MR-PRESSO, MR-RAPS), and conducted multivariable MR to account for correlated metabolites (17–20). We also integrated targeted *in vitro* experiments to explore biological plausibility for priority metabolites.

## 2 Methods and materials

### 2.1 Study design

**Figure 1:**
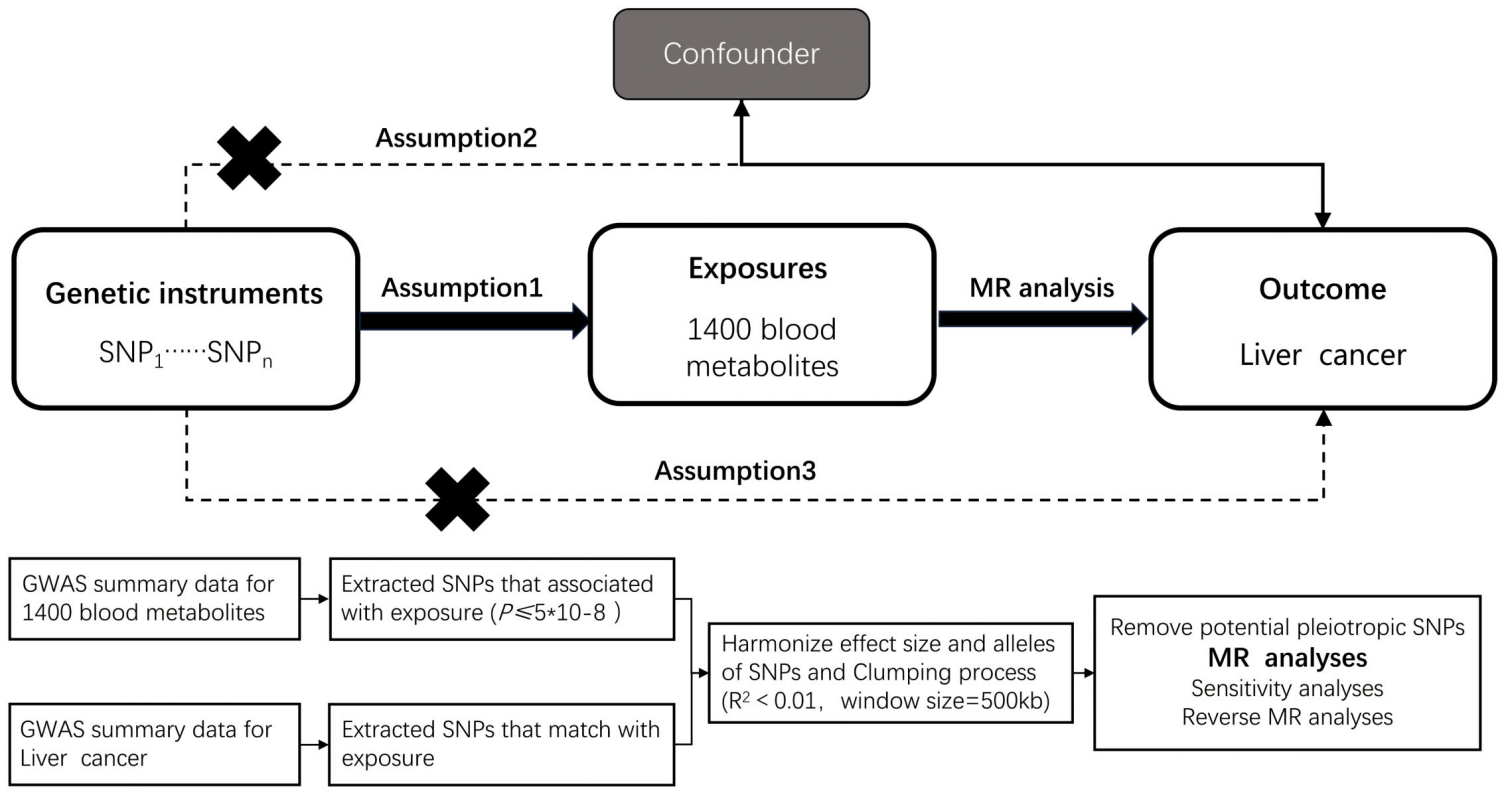
Overview of the design and methods used in this Mendelian randomization study. MR analysis was used to explore the causal relationships, including the following three assumptions: 1. Instrument validity assumption: the genetic variant used as an instrument for the exposure of interest is strongly associated with the exposure but not directly associated with any confounding factors that might influence the outcome. 2. Independence assumption: the genetic variant is independent of any other factors that might influence the outcome, except through its effect on the exposure. 3. Exclusion restriction assumption: the genetic variant affects the outcome only through its effect on the exposure, and not through any other pathways.

Mendelian randomization (MR) inference relies on three core assumptions: (i) relevance (genetic instruments are strongly associated with the exposure), (ii) independence (instruments are independent of confounders), and (iii) exclusion restriction (instruments affect the outcome only via the exposure). We conducted a two-sample MR within a discovery–replication framework (21). The discovery stage used metabolite GWAS (exposures) from the Canadian Longitudinal Study of Aging (CLSA) and outcome GWAS for hepatobiliary malignancies from FinnGen. The replication stage used independent outcome GWAS from the UK Biobank (Neale lab releases) accessed via the IEU OpenGWAS platform, ensuring non-overlapping samples and matched European ancestry; results were combined by meta-analysis. The prespecified workflow comprised: (i) exposure definition and instrument selection, (ii) outcome data ascertainment, (iii) harmonization and instrument diagnostics, (iv) primary and robustness MR estimations, and (v) replication and meta-analysis. A schematic overview is provided in **Figure 1**. Exposure and outcome GWAS primarily include participants of European ancestry; we minimized potential bias from sample overlap by using independent consortia and confirming non-overlap via data source documentation. We acknowledge that the European-ancestry focus may limit generalizability to other populations and highlight this limitation in the Discussion. Reporting followed STROBE-MR guidelines.

### 2.2 Exposure definition and instrument selection

Exposure definition: Circulating metabolite levels (and ratios) were quantified in the CLSA mGWAS by Richards et al. among 8,299 unrelated participants, covering 1,091 metabolites and 309 ratios across amino acids, carbohydrates, cofactors/vitamins, energy-related metabolites, lipids, nucleotides, peptides, and xenobiotics (22). Metabolites with “X-” prefixes denote chemically unidentified features. The GWAS data for plasma metabolites were sourced from the GWAS Catalog (GCST90199621-GCST90201020) (23, 24).

Instrument selection: We selected SNPs associated with each metabolite at genome-wide significance (p < 5×10−8); for sparse traits, a relaxed threshold (p < 1×10−6) was allowed conditional on instrument strength (F > 10). We applied LD clumping using a European LD reference with r2 < 0.001 within a 10,000 kb window to ensure instrument independence, and excluded palindromic SNPs with intermediate allele frequencies. Effect alleles were harmonized across exposure and outcome datasets (25–27).

Instrument strength and directionality: We computed per-exposure F-statistics and applied Steiger filtering to remove variants explaining more variance in the outcome than in the exposure. Summary instrument metrics are provided in **Supplementary Table S1**.

### 2.3 Outcome data

Discovery outcomes: We obtained GWAS summary statistics for hepatobiliary malignancies from FinnGen (release R4; phenotype code C3_LIVER_INTRAHEPATIC_BILE_DUCTS), including 1,046 cases and 10,459 controls. The FinnGen phenotype corresponds to “malignant neoplasm of liver and intrahepatic bile ducts,” primarily based on ICD-10 C22 (malignant neoplasm of liver and intrahepatic bile ducts; including hepatocellular carcinoma and intrahepatic cholangiocarcinoma) and mapped ICD-9 codes. Extrahepatic cholangiocarcinoma (ICD-10 C24.0) and gallbladder cancer (ICD-10 C23) are excluded, as are benign neoplasms. Case/control status and coding followed the FinnGen phenotype documentation.

Replication outcomes: We used the UK Biobank GWAS released by the Neale lab and accessible via the IEU OpenGWAS platform (MRC Integrative Epidemiology Unit, University of Bristol). Specifically, dataset ieu-b-4915 (UK Biobank; 350 cases, 372,016 controls; 7,687,713 SNPs) was analyzed. Summary of GWAS datasets used for outcomes and replication are listed in **Supplementary Table S2**.

The Neale lab UK Biobank GWAS used imputed genotypes from HRC plus UK10K & 1000 Genomes reference panels (GWAS round 2; as released in March 2018; see http://www.nealelab.is/uk-biobank). The corresponding phenotype reflects “malignant neoplasm of liver and intrahepatic bile ducts” derived from ICD-coded hospital records and cancer registries; benign neoplasms and extrahepatic cholangiocarcinoma are not included. To ensure comparability, all outcome effect sizes were harmonized to the log-odds scale prior to MR and meta-analysis. Data sources and accession IDs for all exposure–outcome pairs are listed in **Supplementary Table S3**.

Ethics: Ethical approvals were granted by the original studies (FinnGen Scientific Committee; UK Biobank Ethics Committee for Neale lab analyses). Our MR used de-identified, publicly available summary statistics.

### 2.4 Instrumental variable selection

The study flowchart is presented in **Figure 1**. Circulating plasma metabolites served as exposures and hepatobiliary malignancies as outcomes. For descriptive purposes, metabolites were summarized by chemical classes (e.g., carbohydrates, lipids, amino acids, nucleotides, organic acids, vitamins, hormones, xenobiotics). Instrument selection followed the criteria detailed in Section 2.2 (p-thresholds, LD clumping at r2 < 0.001 within 10,000 kb, MAF ≥ 0.01, allele harmonization, and Steiger filtering). Pleiotropy screening used MR-PRESSO (global and outlier tests) iteratively to identify outliers, complemented by MR-Egger intercept tests. Outliers were removed until the MR-PRESSO global test was non-significant (p > 0.05); the resulting instrument sets were carried forward; to avoid over-correction, we capped removal at a single outlier-deletion step per analysis and retained unfiltered IVW as primary when the global test remained significant, noting that main findings were directionally consistent with and without this filtering.

### 2.5 Statistical analysis

Primary MR estimation used inverse-variance weighted (IVW) models under a random-effects framework when ≥2 instruments were available; for single-instrument exposures, we used the Wald ratio. Robustness estimators included MR-Egger, weighted median, and weighted mode; for sparse or potentially weak-instrument settings, we additionally report MR-RAPS where applicable. Heterogeneity and influence diagnostics included Cochran’s Q, leave-one-out analyses, and Radial MR for outlier detection; when feasible, we performed platform-stratified sensitivity analyses (28, 29). All analyses were conducted in R using TwoSampleMR, MRPRESSO, and RadialMR; meta-analysis employed random-effects models implemented in Review Manager 5.4. Decision criteria for putative causal metabolites were: (1) IVW p < 0.05 with consistent effect directions across robust estimators, (2) no evidence of directional pleiotropy (MR-Egger intercept p ≥ 0.05) and acceptable heterogeneity, (3) stability in leave-one-out and after removing Radial MR/MR-PRESSO outliers, and (4) retained instrument strength (mean F > 10) (28, 30). For binary outcomes, SNP–outcome associations were on the log-odds scale; MR effect estimates are reported as odds ratios per SD increase in metabolite levels, with corresponding 95% confidence intervals.

### 2.6 Replication and meta-analysis

Replication was conducted using the UK Biobank outcome GWAS (Neale lab; IEU OpenGWAS dataset ieu-b-4915), ensuring independence from FinnGen and matched European ancestry. We repeated the harmonization and MR pipeline in the replication dataset. We then combined discovery (FinnGen) and replication (UK Biobank) MR estimates using random-effects inverse-variance–weighted meta-analysis of Wald-type effect estimates on the log-odds scale; between-dataset heterogeneity was assessed via Cochran’s Q and I^2^ (31). When between-dataset heterogeneity was substantial (I^2^ > 50% or Q p < 0.10), we prioritized random-effects results and examined sources of heterogeneity in sensitivity analyses.

### 2.7 Confounding analysis and multivariable MR analysis

To further mitigate confounding via horizontal pleiotropy, we queried PhenoScanner V2 for associations of instruments with hepatobiliary cancer risk factors (alcohol intake, type 2 diabetes, viral hepatitis, medication use, autoimmune traits). Instruments with strong associations (p < 1×10−5) to these traits were excluded and analyses repeated. In addition, we screened instrument sets in IEU OpenGWAS to identify broad pleiotropic signals across common traits and removed discordant instruments in sensitivity analyses. Where instruments were shared across correlated metabolites/classes, we implemented multivariable MR (MVMR-IVW; complemented by MR-PRESSO for outlier correction) to estimate direct effects conditional on correlated exposures. Selection of covariate metabolites in MVMR was guided by biological pathway proximity and phenotypic correlations (32, 33). MVMR models were restricted to instruments available across all included exposures and outcomes in each dataset to preserve sample comparability.

### 2.8 Cell functional experiments

#### 2.8.1 Cell culture and reagents

We employed two human hepatocellular carcinoma (HCC) cell lines: Huh7 and MHCC 97H. All cell lines were obtained from the American Type Culture Collection (ATCC) and the Shanghai Institute of Cell Biology, with confirmation of authenticity using the International Cell Line Authentication Committee’s database (version 8.0) to exclude misidentified lines. Cells were maintained at 37°C with 5% CO2. Huh7 were cultured in high glucose DMEM supplemented with 10% fetal bovine serum (FBS) and 1% penicillin–streptomycin; 97H were cultured in RPMI 1640 with 10% FBS and 1% penicillin–streptomycin unless otherwise stated. No cholangiocarcinoma cell line was included in this study (34–36).

#### 2.8.2 Metabolite treatments and dosing rationale

4-Hydroxyhippurate (4HHA) and 3-hydroxyisobutyrate (3HIB) (purity ≥98%; supplier/catalog) were freshly prepared in sterile culture medium and filtered (0.22 μm).Doses (10, 50, 100 μM) for 4HHA and 3HIB were chosen to bracket reported human circulating levels from upper physiologic to pathophysiologic ranges, with 100 μM for 4HHA explicitly treated as a supra-physiologic sensitivity point. Pilot titrations confirmed the absence of nonspecific cytotoxicity at the chosen ranges (trypan blue exclusion and morphology). Treatments were applied for 24–72 has indicated.

#### 2.8.3 CCK-8 proliferation assay

Cells were seeded in 96-well plates (1×10³ cells/well). After 12 hours, treatments with varying concentrations of 4-hydroxyhippurate and 3-hydroxyisobutyrate were initiated. Vehicle controls received identical culture medium without added metabolites; no DMSO or other organic solvents were used. Positive/assay controls were included as appropriate. At designated time points, 10 μL of CCK-8 solution was added per well, followed by 2-hour incubation. Absorbance at 450 nm (OD_450_) was measured to quantify viability (37).

#### 2.8.4 5-Ethynyl-2’-deoxyuridine proliferation assay

Cell proliferation was assessed using the EdU Apollo567 *In Vitro* Kit (Ribobio, China) according to the manufacturer’s protocol. Briefly, cells were seeded in 6-well plates at a density of 2×10^5^ cells/well. After 12 hours of culture, cells were treated with the specified compounds for 48 hours. Subsequently, cells were incubated with EdU working solution for 2 hours, fixed with 4% paraformaldehyde, permeabilized, and washed. Nuclei were counterstained with 1×Apollo solution and 1×Hoechst 33342. Vehicle controls received the same treatment as above. Fluorescent microscopy images were acquired and analyzed to quantify proliferating cells (38).

#### 2.8.5 Replication and statistical analysis

Each experiment was repeated in at least three independent biological replicates (separate passages/thawed vials), with technical triplicates per condition. Data are presented as mean ± SD unless specified. Two-sided tests were used. For paired, non-normally distributed data we applied the two-sided Wilcoxon signed-rank test; significance thresholds and formats follow the manuscript-wide convention (exact p to three decimals when ≥ 0.001; p < 0.001 otherwise).

## 3 Result

### 3.1 Primary analysis

We first identified 34,843 genome-wide significant SNPs across 1,091 metabolites and 309 ratios. Each filtered instrumental variable contained 12–93 SNPs (4-methyl-2-oxopentanoate levels/3-hydroxylaurate levels represented by 12 SNPs; 2-X15523 showing the largest genetic proxy with 93 SNPs). After LD clumping (r2<0.001, 10,000 kb), harmonization and removal of palindromic SNPs, Steiger filtering, and outlier exclusion by MR-PRESSO/Radial MR, 61 metabolites retained ≥1 valid instrument and proceeded to IVW (or Wald ratio for single-instrument exposures). Detailed data for instrumental variables are presented in **Supplementary Table S1**.

**Figure 2:**
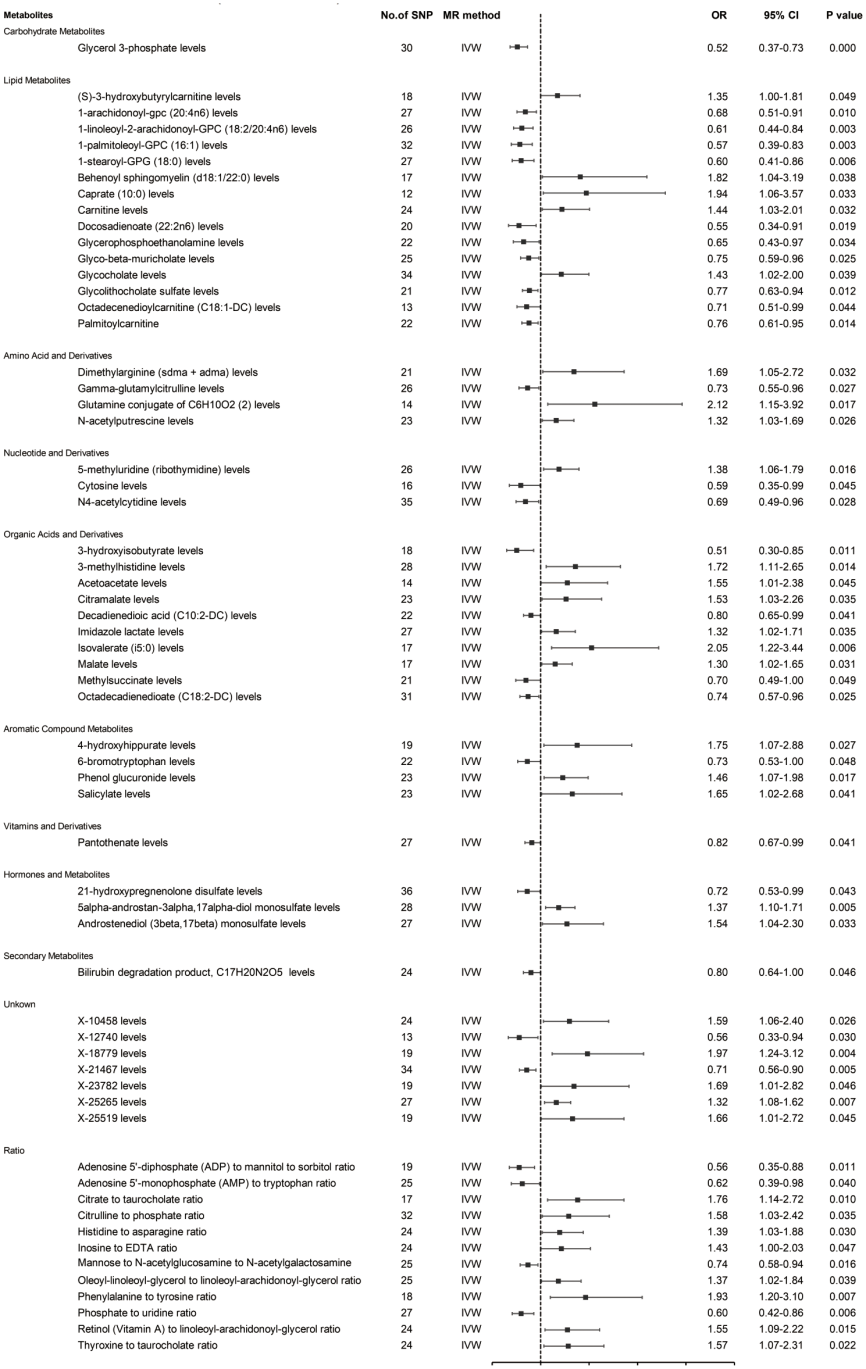
MR estimates (based on IVW) of the effect of blood metabolites on HCC and CCA.

Prior to MR analysis, radial MR was used to identify and remove outliers. Initially, we identified 34,843 SNPs associated with circulating plasma metabolites at genome-wide significance (p < 5×10−8). IVW analysis preliminarily identified 61 metabolites potentially causally associated with hepatobiliary tumors, including 42 known metabolites, 7 unknown metabolites, and 12 metabolite ratios (**Figure 2**).

As shown in **Figure 2**, the 42 known metabolites were categorized by chemical properties into: carbohydrate metabolites, lipid metabolites, amino acids and derivatives, nucleotides and derivatives, organic acids and derivatives, aromatic compounds, vitamins and derivatives, hormones and derivatives, and secondary metabolites.

Following complementary analyses and sensitivity tests, 10 metabolites meeting stringent selection criteria were identified as candidates (**Table 1**), including:

Glycerol 3-phosphate (OR 0.52 95% CI: 0.37-0.73, p < 0.001);
Octadecenedioylcarnitine (C18:1-DC) (OR 0.71, 95% CI: 0.51-0.99, p = 0.044);
Dimethylarginine (sdma + adma) (OR 1.69, 95% CI: 1.05-2.72, p = 0.032);
3-hydroxyisobutyrate (OR 0.51, 95% CI: 0.30-0.85, p = 0.011);
Malate (OR 1.30, 95% CI: 1.02-1.65, p = 0.031);
4-hydroxyhippurate (OR 1.75, 95% CI: 1.07-2.88, p = 0.027);
6-bromotryptophan (OR 0.73, 95% CI: 0.53-1.00, p = 0.048);
5alpha-androstan-3alpha,17alpha-diol monosulfate (OR 1.37, 95% CI: 1.10-1.71, p = 0.005);
X-21467 levels (OR 0.71, 95% CI: 0.56-0.90, p = 0.005);
X-23782 levels (OR 1.69, 95% CI: 1.01-2.82, p = 0.046);

**Table 1:** 10 metabolites meeting stringent selection criteria were identified as candidates.

Metabolites	id.exposure	id.outcome	Study	Cases	Noncases	OR	or_lci95	or_uci95	*P*
Glycerol 3-phosphate levels	GCST90199638	finn-b-C3_LIVER_INTRAHEPATIC_BILE_DUCTS	FinnGen	1046	372016	0.520	0.369	0.731	<0.001
		ieu-b-4915	UK Biobank	350	10459	0.871	0.586	1.885	0.744
Octadecenedioylcarnitine (C18:1-DC) levels	GCST90199970	finn-b-C3_LIVER_INTRAHEPATIC_BILE_DUCTS	FinnGen	1046	372016	0.712	0.511	0.990	0.044
		ieu-b-4915	UK Biobank	350	10459	0.894	0.605	1.184	0.475
Dimethylarginine (sdma + adma) levels	GCST90199832	finn-b-C3_LIVER_INTRAHEPATIC_BILE_DUCTS	FinnGen	1046	372016	1.688	1.046	2.724	0.032
		ieu-b-4915	UK Biobank	350	10459	1.269	0.953	1.699	0.018
3-hydroxyisobutyrate levels	GCST90200308	finn-b-C3_LIVER_INTRAHEPATIC_BILE_DUCTS	FinnGen	1046	372016	0.507	0.300	0.855	0.011
		ieu-b-4915	UK Biobank	350	10459	0.444	0.160	0.788	0.008
Malate levels	GCST90200398	finn-b-C3_LIVER_INTRAHEPATIC_BILE_DUCTS	FinnGen	1046	372016	1.300	1.024	1.652	0.031
		ieu-b-4915	UK Biobank	350	10459	1.016	0.977	1.056	0.942
4-hydroxyhippurate levels	GCST90199765	finn-b-C3_LIVER_INTRAHEPATIC_BILE_DUCTS	FinnGen	1046	372016	1.754	1.068	2.880	0.027
		ieu-b-4915	UK Biobank	350	10459	1.466	1.107	1.750	0.028
6-bromotryptophan levels	GCST90200201	finn-b-C3_LIVER_INTRAHEPATIC_BILE_DUCTS	FinnGen	1046	372016	0.726	0.528	0.997	0.048
		ieu-b-4915	UK Biobank	350	10459	0.999	0.755	1.242	0.203
5alpha-androstan-3alpha,17alpha-diol monosulfate levels	GCST90199850	finn-b-C3_LIVER_INTRAHEPATIC_BILE_DUCTS	FinnGen	1046	372016	1.373	1.103	1.709	0.005
		ieu-b-4915	UK Biobank	350	10459	1.058	0.735	1.382	0.724
X-21467 levels	GCST90200594	finn-b-C3_LIVER_INTRAHEPATIC_BILE_DUCTS	FinnGen	1046	372016	0.713	0.563	0.903	0.005
		ieu-b-4915	UK Biobank	350	10459	0.990	0.721	1.259	0.942
X-23782 levels	GCST90200618	finn-b-C3_LIVER_INTRAHEPATIC_BILE_DUCTS	FinnGen	1046	372016	1.689	1.010	2.823	0.046
		ieu-b-4915	UK Biobank	350	10459	1.041	0.385	1.698	0.902

with consistent directions and magnitudes observed across IVW, MR-Egger, and weighted median (WM) estimates (**Figure 3**). Both Cochran’s Q test (p > 0.05) and MR-Egger intercept test (p > 0.05) provided strong evidence against heterogeneity and pleiotropy (**Supplementary Table S4**). Leave-one-out (LOO) analysis further confirmed that no single SNP disproportionately influenced the MR estimates (**Supplementary Figure S1**). **Supplementary Figure S2** presents forest plots of Mendelian randomization effect estimates, displaying both IVW and MR-Egger results for traits with significant IVW associations. These 10 blood metabolites were thus considered robust candidates for subsequent analyses.

**Figure 3:**
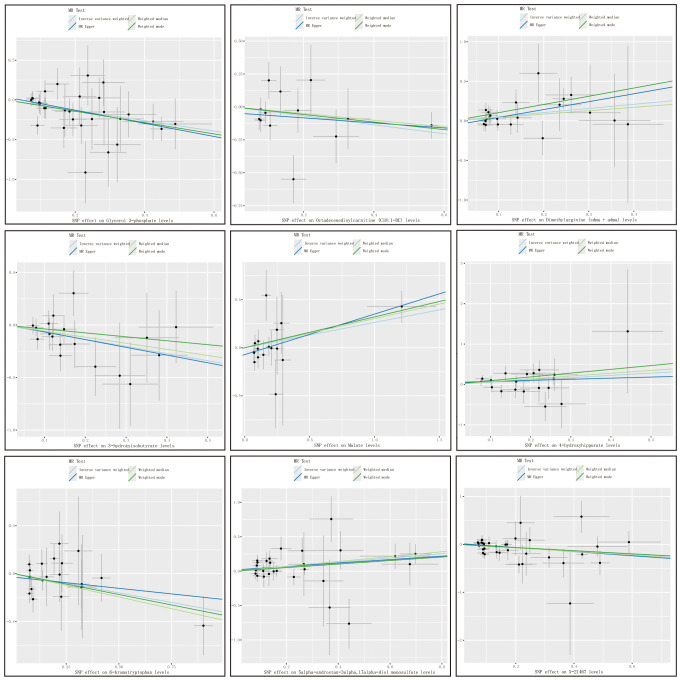
Metabolite scatter plots with a forward direction. Vertical axis: The effect value of SNP on HCC and CAA; Horizontal axis: The affect value of SNP on different metabolites; Colored lines represent the results of MR analysis based on four methods.

### 3.2 Replication, meta-analysis and MVMR

**Figure 4:**
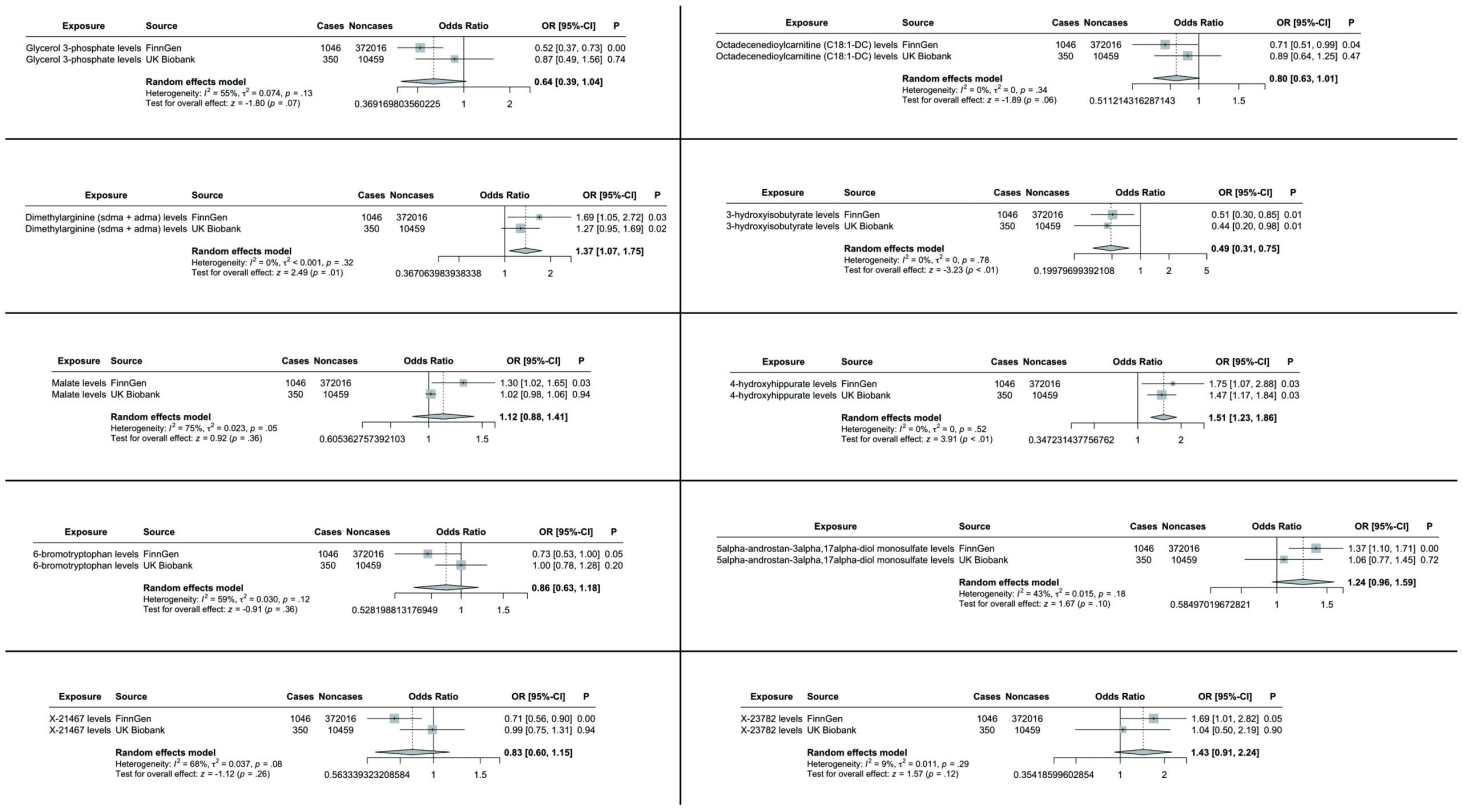
TSMR analysis and meta-analysis of the relationship between blood metabolites and HCC and CCA. The reported values were calculated by the IVW method. TSMR, two-sample Mendelian randomization.

To enhance the robustness of our findings, we replicated the MR analysis using an independent GWAS dataset for hepatobiliary tumors. As anticipated, similar trends were observed in this validation cohort. Meta-analysis of both datasets conclusively identified three blood metabolites significantly influencing hepatobiliary malignancies (**Figure 4**).

Specifically, elevated levels of dimethylarginine (SDMA + ADMA) (OR 1.37, 95% CI: 1.07-1.75, p = 0.01) and 4-hydroxyhippurate (OR 1.51, 95% CI: 1.23-1.86, p < 0.01) were associated with increased tumor risk, while 3-hydroxyisobutyrate (OR 0.49, 95% CI: 0.31-0.75, p < 0.01) demonstrated protective effects. The remaining candidate metabolites showed non-significant associations in the combined meta-analysis (**Figure 4**).

**Figure 5:**
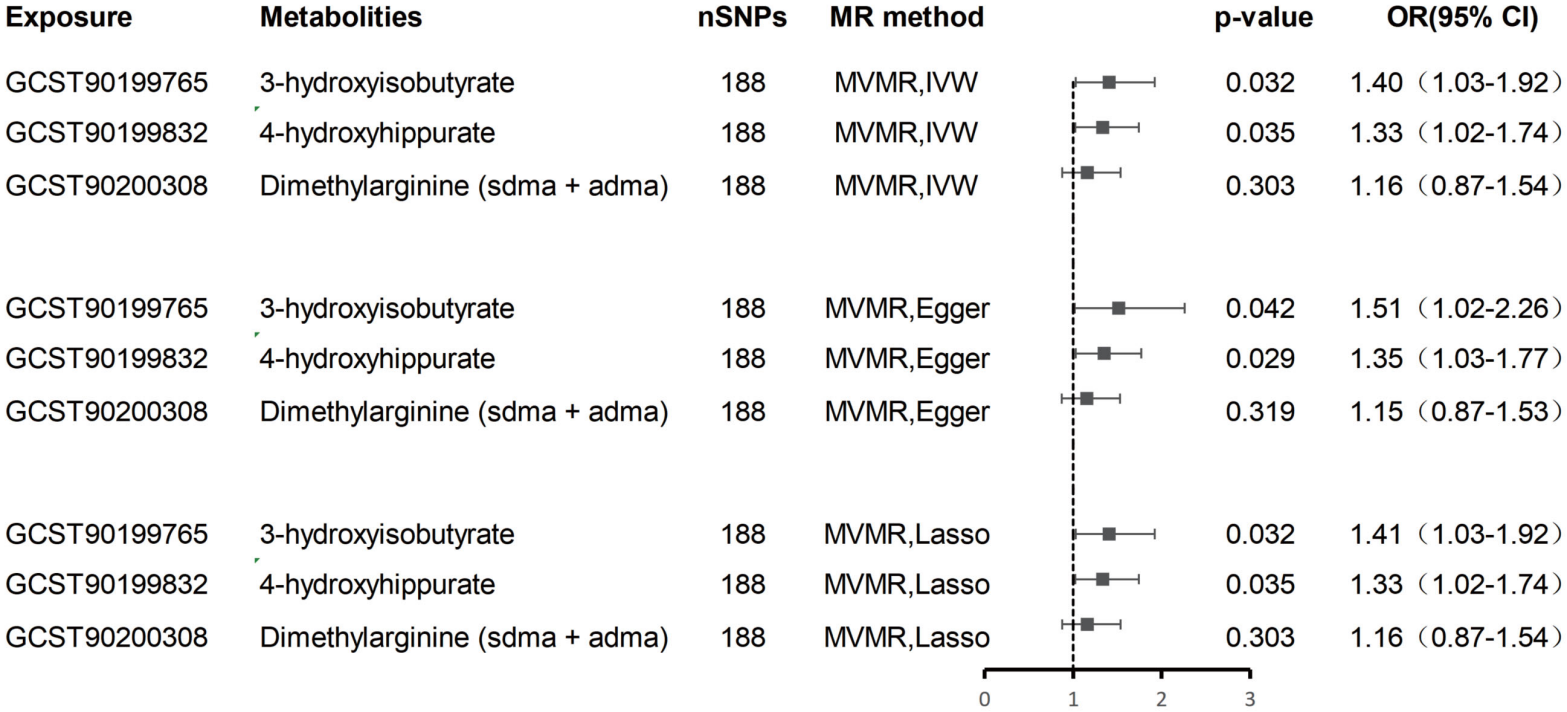
Multivariate MR analysis of the direct effect of 3-hydroxyisobutyrate, 4-hydroxyhippurate, and Dimethylarginine on hepatobiliary malignancies.

MVMR analyses adjusting for metabolite interdependencies - employing both IVW and MR-PRESSO approaches (**Figure 5**) - confirmed that genetically predicted 4-hydroxyhippurate and 3-hydroxyisobutyrate exert direct, independent effects on hepatobiliary malignancy risk, unaffected by other metabolic factors. **Supplementary Table S4** details heterogeneity indices and pleiotropy tests arising from the MVMR models, demonstrating acceptable heterogeneity and no significant pleiotropy.

### 3.3 Cell functional experiments

**Figure 6:**
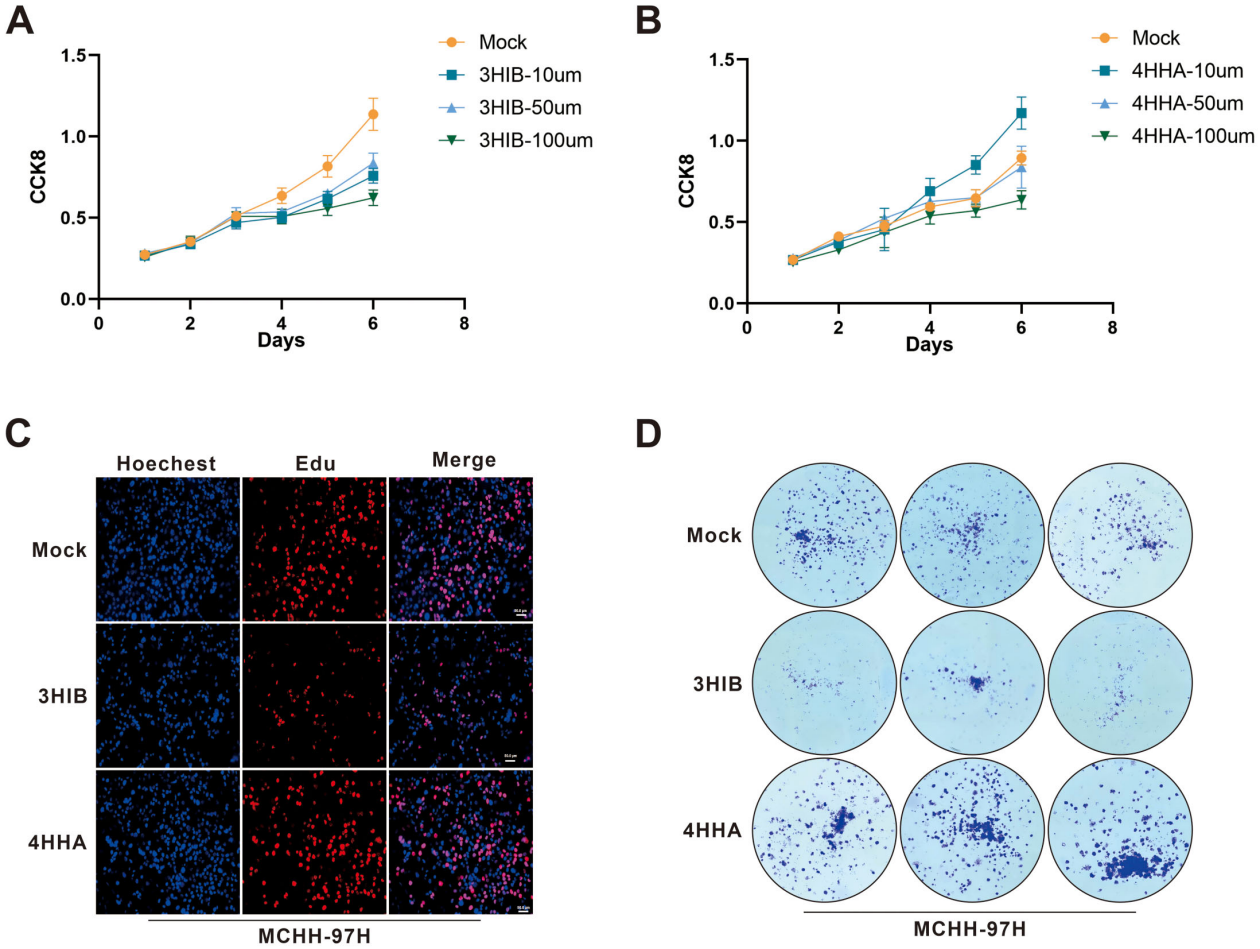
Functional cellular assays validated the tumor-suppressive effect of 3HIB and the tumor-promoting effect of 4HHA. **(A, B)**. 3HIB inhibited hepatocellular carcinoma cell proliferation at all tested concentrations, whereas 10 µM 4HHA enhanced proliferation. **(C, D)**. Both EdU and colony formation assays demonstrated that 50 μM 3HIB suppressed proliferation, while 10 µM 4HHA promoted it.

*In vitro* experiments demonstrated that 4-hydroxyhippurate (4HHA) promoted hepatocellular carcinoma cell proliferation, while 3-hydroxyisobutyrate (3HIB) exerted inhibitory effects (**Figure 6**). Specifically, the CCK-8 assays showed that 3HIB reduced cell viability across all tested concentrations in Huh7 (**Figure 6A**) and MHCC-97H cells (p =0.0313), whereas 10 µM 4HHA increased viability in both lines (**Figure 6B**).

Consistent with these findings, the EdU incorporation assay (**Figure 6C**) indicated that 50 µM 3HIB suppressed hepatoma cell proliferation, while 10 µM 4HHA enhanced proliferative capacity; similarly, the colony formation assay (**Figure 6D**) revealed fewer colonies following treatment with 50 µM 3HIB and increased colony numbers with 10 µM 4HHA.

## 4 Discussion

Based on a comprehensive metabolome-wide Mendelian randomization analysis, this study employed a two-sample Mendelian randomization (TSMR) approach to systematically evaluate potential causal relationships between 1,400 serum metabolites and the risk of hepatobiliary and cholangiocellular malignancies (22). The results identified 10 blood metabolites potentially influencing the occurrence of hepatobiliary and cholangiocellular malignancies, which were further validated in an independent dataset (22, 27, 30). A meta-analysis confirmed that three of these blood metabolites exhibited significant effects on hepatobiliary and cholangiocellular malignancies. Given the biological heterogeneity across hepatobiliary malignancies, our results pertain to liver and intrahepatic bile duct cancers rather than the entire hepatobiliary spectrum. For the positive findings in TSMR analysis, multivariable Mendelian randomization (MVMR) was performed to adjust for potential confounding factors, revealing that 4-hydroxyhippurate and 3-hydroxyisobutyrate could directly affect hepatobiliary malignancies independently of other metabolites (39–42). Finally, cellular experiments were conducted to validate their biological functions.

This study design not only enhances the accuracy of causal inference but also provides direct experimental evidence for the mechanistic involvement of metabolites in the pathogenesis of hepatobiliary and cholangiocellular malignancies. The findings establish a foundation for a deeper understanding of the metabolic regulatory networks underlying these malignancies. The application of multivariable Mendelian randomization (MVMR) analysis effectively addresses the limitations of conventional univariable approaches in failing to consider metabolic pathway complexity (33).

As a gut microbiota–derived polyphenol metabolite, 4-hydroxyhippurate (4HHA) showed an independent positive association with hepatobiliary/cholangiocellular malignancies after adjusting for related metabolites (OR 1.75, 95% CI: 1.07–2.88, p = 0.027) (43). 3-Hydroxyisobutyrate (3-HIB), a valine-catabolism intermediate, showed a strengthened protective association after adjusting for BCAAs and related acylcarnitines (OR 0.51, 95% CI: 0.30–0.85, p = 0.011) (44). These findings reinforce colorectal-cancer literature that independent metabolite effects require correction for pathway collinearity, underscoring multivariable modeling in metabolic-network analyses (45).

4HHA is a hydroxylated hippurate formed via microbial polyphenol metabolism plus hepatic conjugation, engaging the gut–liver–kidney axis and serving as a urinary biomarker (46). In high-fat-diet models, dysbiosis–LPS–TLR4/NF-κB activation promotes steatosis and inflammation, creating an HCC-permissive milieu; while no direct 4HHA–CCA/HCC link is reported, gut–liver–immune mechanisms (e.g., biliary epithelial inflammation, angiogenesis) are plausible and testable (12). 3-HIB arises mainly from valine (possibly thymine) catabolism across liver, muscle, and kidney; inborn errors can cause accumulation (e.g., 3-hydroxyisobutyric aciduria) (44). HIBADH is upregulated during hepatocyte injury and may affect mitochondrial energy metabolism (e.g., ATP production) (42, 47). Direct evidence in biliary/hepatic tumors is lacking, but modulation via the gut microbiota–immune microenvironment axis is a reasonable hypothesis, analogous to bile-acid–mediated effects (48, 49).

In evaluating the validity of the Mendelian randomization (MR) analysis results, we primarily focused on pleiotropy, particularly horizontal pleiotropy—where genetic variants influence hepatobiliary malignancies through pathways other than serum metabolites. In this study, we first used PhenoScanner to identify and remove single-nucleotide polymorphisms (SNPs) potentially associated with alternative pleiotropic pathways. Second, we employed MR-Egger regression, the weighted median method, and MR-PRESSO to address pleiotropy (21, 50). Although the estimates generated by these methods showed slight variations, their conclusions were consistent, with none indicating significant pleiotropy, demonstrating the robustness of our findings across different approaches.

Additionally, while the multivariable Mendelian randomization (MVMR) analysis adjusted for several known confounding factors, there may still exist unrecognized or unmeasured confounders that simultaneously influence both the exposure and outcome, potentially introducing bias into the causal estimates (32, 33, 51). However, no evidence of horizontal pleiotropy was detected in this study, suggesting that the observed causal associations are not significantly affected by confounding factors.

This study possesses several notable strengths. The primary advantage lies in its Mendelian randomization (MR) design, which substantially mitigates the influence of confounding factors and reverse causation. Secondly, we employed multivariable Mendelian randomization (MVMR) analysis to adjust for residual confounding, thereby enhancing the reliability of causal inferences between serum metabolites and hepatobiliary malignancy risk. Furthermore, we conducted functional cellular validation experiments on the identified metabolites to reinforce the robustness of the causal relationships. Additionally, the utilization of multiple independent datasets in this study effectively reduced potential biases arising from population stratification.

This study has several limitations. The metabolite ratios we analyzed were precomputed by the source mGWAS (not defined by us); while some may proxy pathway balance, many are statistical constructs with limited mechanistic interpretability due to shared determinants or scaling. We therefore emphasize ratio signals consistent with component metabolites, known biochemistry, or independent evidence, and de-emphasize those showing inconsistency or pleiotropy. To minimize sample overlap bias, we selected GWAS data from different sources and populations, but potential overlap may persist due to large, concentrated sample sizes. However, in TSMR analysis, strongly associated SNPs (all F-statistics≥10) were selected, suggesting minimal bias from sample overlap (51). First, a lenient threshold (p<5×10^-6^) was used to include more SNPs, which improved statistical power but may increase pleiotropy risk, so we conducted stringent sensitivity analyses and controlled for pleiotropic pathways, with all SNPs’ F-statistics≥10 ensuring strong instrument-exposure associations. Second, stratified analyses by age, sex, or tumor stage were impossible due to GWAS data limitations, pointing to future research directions (26, 52, 53). Methodologically, MR relies on GWAS-identified genetic variants, and underpowered GWAS may cause bias; moreover, MR assumes no gene-environment interactions, so if environmental factors modulate genetic effects on exposures/outcomes, true causality may be misestimated. Furthermore, we acknowledge that the inclusion of “X-” (unknown) metabolite features, while minimizing annotation bias, limits mechanistic interpretability and translational relevance in the absence of chemical identification, and thus these findings should be viewed as hypothesis-generating pending targeted annotation and validation. Thus, our study mainly provides genetic-level evidence and suggests more prospective studies. Experimentally, only the cell proliferation effects of two metabolites were tested without mechanistic studies or microenvironment simulation, lacking animal validation; single-concentration cell experiments require dose-response tests to determine effect thresholds; MR captures static genetic associations without considering dynamic influences like diet/circadian rhythm on 4HHA/3HIB levels.

In subsequent studies, we will focus on addressing the current limitations by conducting cellular experiments to investigate specific regulatory sites and signaling pathways, dynamically monitoring metabolomic changes during carcinogenesis, and jointly validating their potential value for early cancer screening.

## 5 Conclusion

In summary, this study has innovatively identified and validated two key metabolites associated with hepatobiliary tumors through methodological advancements. These findings not only provide novel insights into the etiological research of hepatobiliary tumors but also establish a foundation for developing metabolism-based early diagnostic biomarkers and therapeutic targets. The results highlight the pivotal role of metabolites in the prevention and treatment of hepatobiliary tumors, underscoring that translating these fundamental discoveries into clinical applications will represent a crucial direction for future research.

## Data Availability

The original contributions presented in the study are included in the article/**Supplementary Material**. Further inquiries can be directed to the corresponding authors.

## References

[1] Sung H Ferlay J Siegel RL Laversanne M Soerjomataram I Jemal A. Global cancer statistics 2020: GLOBOCAN estimates of incidence and mortality worldwide for 36 cancers in 185 countries. CA: A Cancer J Clin. (2021) 71:209–49. doi: 10.3322/caac.21660, PMID: 33538338

[2] Bruix J Gores GJ Mazzaferro V. Hepatocellular carcinoma: clinical frontiers and perspectives. Gut. (2014) 63:844–55. doi: 10.1136/gutjnl-2013-306627, PMID: 24531850 PMC4337888

[3] Banales JM Marin JJG Lamarca A Rodrigues PM Khan SA Roberts LR. Cholangiocarcinoma 2020: the next horizon in mechanisms and management. Nat Rev Gastroenterol Hepatol. (2020) 17:557–88. doi: 10.1038/s41575-020-0310-z, PMID: 32606456 PMC7447603

[4] Younossi ZM Koenig AB Abdelatif D Fazel Y Henry L Wymer M. Global epidemiology of nonalcoholic fatty liver disease—Meta-analytic assessment of prevalence, incidence, and outcomes. Hepatology. (2016) 64:73. doi: 10.1002/hep.28431, PMID: 26707365

[5] Reig M Forner A Rimola J Ferrer-Fàbrega J Burrel M Garcia-Criado Á. BCLC strategy for prognosis prediction and treatment recommendation: The 2022 update. J Hepatol. (2022) 76:681–93. doi: 10.1016/j.jhep.2021.11.018, PMID: 34801630 PMC8866082

[6] Pavlova NN Thompson CB. The emerging hallmarks of cancer metabolism. Cell Metab. (2016) 23:27–47. doi: 10.1016/j.cmet.2015.12.006, PMID: 26771115 PMC4715268

[7] De Matteis S Ragusa A Marisi G De Domenico S Casadei Gardini A Bonafè M. Aberrant metabolism in hepatocellular carcinoma provides diagnostic and therapeutic opportunities. Oxid Med Cell Longev. (2018) 2018:7512159. doi: 10.1155/2018/7512159, PMID: 30524660 PMC6247426

[8] Ponziani FR Bhoori S Castelli C Putignani L Rivoltini L Del Chierico F. Hepatocellular carcinoma is associated with gut microbiota profile and inflammation in nonalcoholic fatty liver disease. Hepatology. (2019) 69:107. doi: 10.1002/hep.30036, PMID: 29665135

[9] Pallister T Jackson MA Martin TC Zierer J Jennings A Mohney RP. Hippurate as a metabolomic marker of gut microbiome diversity: Modulation by diet and relationship to metabolic syndrome. Sci Rep. (2017) 7:13670. doi: 10.1038/s41598-017-13722-4, PMID: 29057986 PMC5651863

[10] Chiang JYL Ferrell JM. Bile acids as metabolic regulators and nutrient sensors. Annu Rev Nutr. (2019) 39:175–200. doi: 10.1146/annurev-nutr-082018-124344, PMID: 31018107 PMC6996089

[11] Hu S Lin Z Hu M-J Tan J-S Guo T-T Huang X. Causal relationships of circulating amino acids with cardiovascular disease: a trans-ancestry Mendelian randomization analysis. J Transl Med. (2023) 21:699. doi: 10.1186/s12967-023-04580-y, PMID: 37805555 PMC10559604

[12] Zhu K Li R Qiu Z Yu H Xu K Li R. Circulating bile acids, bile acids receptor polymorphisms, and risk of chronic kidney disease among individuals with newly diagnosed type 2 diabetes: A prospective study. Am J Clin Nutr. (2025) 122(3):841–52. doi: 10.1016/j.ajcnut.2025.07.017, PMID: 40716521

[13] Fleishman JS Kumar S. Bile acid metabolism and signaling in health and disease: molecular mechanisms and therapeutic targets. Signal Transduct Target Ther. (2024) 9:97. doi: 10.1038/s41392-024-01811-6, PMID: 38664391 PMC11045871

[14] Lotta LA Pietzner M Stewart ID Wittemans LBL Li C Bonelli R. Cross-platform genetic discovery of small molecule products of metabolism and application to clinical outcomes. Nat Genet. (2021) 53:54–64. doi: 10.1038/s41588-020-00751-5, PMID: 33414548 PMC7612925

[15] Davies NM Holmes MV Davey Smith G. Reading Mendelian randomisation studies: a guide, glossary, and checklist for clinicians. BMJ. (2018) 362:k601. doi: 10.1136/bmj.k601, PMID: 30002074 PMC6041728

[16] Hernán MA Robins JM. Using big data to emulate a target trial when a randomized trial is not available. Am J Epidemiol. (2016) 183:758–64. doi: 10.1093/aje/kwv254, PMID: 26994063 PMC4832051

[17] Verbanck M Chen C-Y Neale B Do R. Publisher Correction: Detection of widespread horizontal pleiotropy in causal relationships inferred from Mendelian randomization between complex traits and diseases. Nat Genet. (2018) 50:1196. doi: 10.1038/s41588-018-0164-2, PMID: 29967445

[18] Hartwig FP Davey Smith G Bowden J. Robust inference in summary data Mendelian randomization via the zero modal pleiotropy assumption. Int J Epidemiol. (2017) 46:1985–98. doi: 10.1093/ije/dyx102, PMID: 29040600 PMC5837715

[19] Xu Y Ritchie SC Liang Y Timmers PRHJ Pietzner M Lannelongue L. An atlas of genetic scores to predict multi-omic traits. Nature. (2023) 616:123–31. doi: 10.1038/s41586-023-05844-9, PMID: 36991119 PMC10323211

[20] Bycroft C Freeman C Petkova D Band G Elliott LT Sharp K. The UK Biobank resource with deep phenotyping and genomic data. Nature. (2018) 562:203–9. doi: 10.1038/s41586-018-0579-z, PMID: 30305743 PMC6786975

[21] Cai Y Jia X Xu L Chen H Xie S Cai J. Interleukin-17 and inflammatory bowel disease: a 2-sample Mendelian randomization study. Front Immunol. (2023) 14:1238457. doi: 10.3389/fimmu.2023.1238457, PMID: 38045694 PMC10690942

[22] Lu X Chen Y Jiang Y Ning J Liu S Lv Z. Genetically predicted 1400 blood metabolites in relation to risk of prostate cancer: A mendelian randomization study. Aging Med. (2025) 8:249–57. doi: 10.1002/agm2.70016, PMID: 40620513 PMC12226420

[23] Chen Y Lu T Pettersson-Kymmer U Stewart ID Butler-Laporte G Nakanishi T. Genomic atlas of the plasma metabolome prioritizes metabolites implicated in human diseases. Nat Genet. (2023) 55:44–53. doi: 10.1038/s41588-022-01270-1, PMID: 36635386 PMC7614162

[24] Bouras E Karhunen V Gill D Huang J Haycock PC Gunter MJ. Circulating inflammatory cytokines and risk of five cancers: a Mendelian randomization analysis. BMC Med. (2022) 20:3. doi: 10.1186/s12916-021-02193-0, PMID: 35012533 PMC8750876

[25] Petrisor A Stanescu AMA Papacocea IR Panaitescu E Peagu R Moldoveanu AC. Non-invasive laboratory, imaging and elastography markers in predicting varices with high risk of bleeding in cirrhotic patients. Rom J Intern Med. (2021) 59:194–200. doi: 10.2478/rjim-2021-0001, PMID: 33544557

[26] Au Yeung SL Gill D. Standardizing the reporting of Mendelian randomization studies. BMC Med. (2023) 21:187. doi: 10.1186/s12916-023-02894-8, PMID: 37198682 PMC10193619

[27] Cao D Zhang Y Zhang S Li J Yang Q Wang P. Risk of Alzheimer’s disease and genetically predicted levels of 1400 plasma metabolites: a Mendelian randomization study. Sci Rep. (2024) 14:26078. doi: 10.1038/s41598-024-77921-6, PMID: 39478193 PMC11525545

[28] Chen J-H Zeng L-Y Zhao Y-F Tang H-X Lei H Wan Y-F. Causal effects of gut microbiota on sepsis: a two-sample Mendelian randomization study. Front Microbiol. (2023) 14:1167416. doi: 10.3389/fmicb.2023.1167416, PMID: 37234519 PMC10206031

[29] Flatby HM Ravi A Damås JK Solligård E Rogne T. Circulating levels of micronutrients and risk of infections: a Mendelian randomization study. BMC Med. (2023) 21:84. doi: 10.1186/s12916-023-02780-3, PMID: 36882828 PMC9993583

[30] Gray N Lawler NG Yang R Morillon A-C Gay MCL Bong S-H. A simultaneous exploratory and quantitative amino acid and biogenic amine metabolic profiling platform for rapid disease phenotyping via UPLC-QToF-MS. Talanta. (2021) 223:121872. doi: 10.1016/j.talanta.2020.121872, PMID: 33298292

[31] Feng Q Grant AJ Yang Q Burgess S Bešević J Conroy M. Genetically predicted vegetable intake and cardiovascular diseases and risk factors: an investigation with mendelian randomization. Nutrients. (2023) 15:3682. doi: 10.3390/nu15173682, PMID: 37686714 PMC10490460

[32] Kintu C Soremekun O Kamiza AB Kalungi A Mayanja R Kalyesubula R. The causal effects of lipid traits on kidney function in Africans: bidirectional and multivariable Mendelian-randomization study. eBioMedicine. (2023) 90:104537. doi: 10.1016/j.ebiom.2023.104537, PMID: 37001235 PMC10070509

[33] Sanderson E. Multivariable mendelian randomization and mediation. Cold Spring Harb Perspect Med. (2021) 11:a038984. doi: 10.1101/cshperspect.a038984, PMID: 32341063 PMC7849347

[34] Li X Gao Y Cheng T Lin J Lou S Wang L. Genetic modulation of lncPSMB1 confers non-syndromic cleft lip with or without cleft palate susceptibility by promoting cell apoptosis. Commun Biol. (2025) 8:1123. doi: 10.1038/s42003-025-08563-1, PMID: 40730884 PMC12307944

[35] Tokumura K Fukasawa K Ichikawa J Sadamori K Hiraiwa M Hinoi E. PDK1-dependent metabolic reprogramming regulates stemness and tumorigenicity of osteosarcoma stem cells through ATF3. Cell Death Dis. (2025) 16:574. doi: 10.1038/s41419-025-07903-7, PMID: 40730548 PMC12307947

[36] Pharmacological CLK inhibition disrupts SR protein function and RNA splicing blocking cell growth and migration in TNBC | Breast Cancer Research | Full Text . 10.1186/s13058-025-02091-w PMC12309053 40731028

[37] Harmine inhibits oxidative phosphorylation, thus regulating the polarization of macrophages mediated by extracellular adenosine in endometriosis | Human Reproduction . Oxford Academic. 10.1093/humrep/deaf130 40730246

[38] Shen J Wang W Chen L Xu L Huang X Tan Y. SIRT1 alleviates hyperglycemia-induced oxidative stress and dysfunction of limbal niche cells by deacetylating FOXO1. Free Radical Biol Med. (2025) 239:130–44. doi: 10.1016/j.freeradbiomed.2025.07.032, PMID: 40714155

[39] Chan Q Wren GM Lau C-HE Ebbels TMD Gibson R Loo RL. Blood pressure interactions with the DASH dietary pattern, sodium, and potassium: The International Study of Macro-/Micronutrients and Blood Pressure (INTERMAP). Am J Clin Nutr. (2022) 116:216–29. doi: 10.1093/ajcn/nqac067, PMID: 35285859 PMC9257466

[40] Blümlhuber A Freuer D Wawro N Rohm F Meisinger C Linseisen J. Association between habitual dietary intake and urinary metabolites in adults—Results of a population-based study. Metabolites. (2025) 15:441. doi: 10.3390/metabo15070441, PMID: 40710542 PMC12297984

[41] Bjune MS Lawrence-Archer L Laupsa-Borge J Sommersten CH McCann A Glastad RC. Metabolic role of the hepatic valine/3-hydroxyisobutyrate (3-HIB) pathway in fatty liver disease. eBioMedicine. (2023) 91:104569. doi: 10.1016/j.ebiom.2023.104569, PMID: 37084480 PMC10148099

[42] Meyer M Hollenbeck JC Reunert J Seelhöfer A Rust S Fobker M. 3-Hydroxyisobutyrate dehydrogenase (HIBADH) deficiency—A novel disorder of valine metabolism. J Inherited Metab Dis. (2021) 44:1323–9. doi: 10.1002/jimd.12410, PMID: 34176136

[43] Sun S Jiao M Han C Zhang Q Shi W Shi J. Causal effects of genetically determined metabolites on risk of polycystic ovary syndrome: A mendelian randomization study. Front Endocrinol (Lausanne). (2020) 11:621. doi: 10.3389/fendo.2020.00621, PMID: 33013699 PMC7505923

[44] Che L Liu L Xu M Fan Z Niu L Chen Y. Valine metabolite, 3-hydroxyisobutyrate, promotes lipid metabolism and cell proliferation in porcine mammary gland epithelial cells. Front Nutr. (2025) 11:1524738. doi: 10.3389/fnut.2024.1524738, PMID: 39867557 PMC11757131

[45] Patil N Mirveis Z Byrne HJ. Monitoring cellular glycolysis pathway kinetics in the extracellular medium using label-free, Raman spectroscopy. Spectrochim Acta Part A: Mol Biomol Spectrosc. (2025) 340:126363. doi: 10.1016/j.saa.2025.126363, PMID: 40349395

[46] Mero S Satolli S Galatolo D Canto FD Armando M Astrea G. HPDL biallelic variants in cerebral palsy and childhood-onset hereditary spastic paraplegia: human and zebrafish insights. Movement Disord. (2025). doi: 10.1002/mds.30296, PMID: 40719007

[47] Sasarman F Ferdinandusse S Sinasac DS Fung E Sparkes R Reeves M. 3-Hydroxyisobutyric acid dehydrogenase deficiency: Expanding the clinical spectrum and quantitation of D- and L-3-Hydroxyisobutyric acid by an LC–MS/MS method. J Inherited Metab Dis. (2022) 45:445–55. doi: 10.1002/jimd.12486, PMID: 35174513

[48] Wang J Cheng W Yang R. Nervous system–gut microbiota–immune system axis: future directions for preventing tumor. Front Immunol. (2025) 16:1535955. doi: 10.3389/fimmu.2025.1535955, PMID: 40376000 PMC12078214

[49] Jing Z Yinhang W Jian C Zhanbo Q Xinyue W Shuwen H. Interaction between gut microbiota and T cell immunity in colorectal cancer. Autoimmun Rev. (2025) 24:103807. doi: 10.1016/j.autrev.2025.103807, PMID: 40139455

[50] Bowden J Del Greco MF Minelli C Davey Smith G Sheehan NA Thompson JR. Assessing the suitability of summary data for two-sample Mendelian randomization analyses using MR-Egger regression: the role of the I2 statistic. Int J Epidemiol. (2016) 45:1961–74. doi: 10.1093/ije/dyw220, PMID: 27616674 PMC5446088

[51] Rasooly D Peloso GM. Two-sample multivariable mendelian randomization analysis using R. Curr Protoc. (2021) 1:e335. doi: 10.1002/cpz1.335, PMID: 34936225 PMC8767787

[52] Lv Y Cheng X Dong Q. SGLT1 and SGLT2 inhibition, circulating metabolites, and cerebral small vessel disease: a mediation Mendelian Randomization study. Cardiovasc Diabetol. (2024) 23:157. doi: 10.1186/s12933-024-02255-6, PMID: 38715111 PMC11077823

[53] Näslund-Koch C Vedel-Krogh S Bojesen SE Skov L. Smoking is an independent but not a causal risk factor for moderate to severe psoriasis: A Mendelian randomization study of 105,912 individuals. Front Immunol. (2023) 14:1119144. doi: 10.3389/fimmu.2023.1119144, PMID: 36911745 PMC9992829

