# Supplementary material for: Genetically instrumented circulating metabolites and hepatobiliary cancer risk: A multi-tiered Mendelian randomization and functional interrogation
Source: Front Oncol. 2025 Oct 27;15:1680865. doi: 10.3389/fonc.2025.1680865 (PMC12597744; doi:10.3389/fonc.2025.1680865)
Supplement: Supplementary file 2 [file DataSheet2.docx]

**Supplementary Materials**

**Genetically Instrumented Circulating Metabolites and Hepatobiliary Cancer Risk: A Multi-tiered Mendelian Randomization and Functional Interrogation**

**Supplementary Figure**

Supplementary Figure 2: Forest plots for the Mendelian randomization (MR) effect analysis (MR Egger and IVW) of the significant inverse variance weighted (IVW) estimates.
